# Left Atrioventricular Coupling Index in Feline Hypertrophic Cardiomyopathy: Association with Disease Severity and Arterial Thromboembolism

**DOI:** 10.3390/vetsci13050491

**Published:** 2026-05-19

**Authors:** Tuğba Varlik, Didem Algan, Ryou Tanaka, Zeki Yilmaz

**Affiliations:** 1 Department of Internal Medicine, Veterinary Faculty, Bursa Uludag University, 16059 Bursa, Türkiye; tugbav.vet@gmail.com (T.V.); didem.algann@gmail.com (D.A.); 2 Department of Veterinary Medicine, Faculty of Agriculture, Tokyo University of Agriculture and Technology, Fuchu 183-8538, Japan; fu0253@go.tuat.ac.jp

**Keywords:** atrioventricular coupling, cats, echocardiography, hypertrophic cardiomyopathy, LACI, thromboembolism

## Abstract

HCM is the most prevalent cardiac disease in cats and is associated with substantial morbidity and mortality. Among its complications, FATE represents a major adverse clinical outcome. Conventional echocardiographic indices typically assess atrial or ventricular parameters separately and may not fully capture the structural relationship between the LA and LV. The left atrioventricular coupling index (LACI) is a volumetric ratio that combines LA remodeling and LV chamber size at end-diastole into a single structural index. This retrospective, cross-sectional observational study included 91 cats, classified according to ACVIM guidelines into healthy controls (*n* = 33), asymptomatic HCM (stage B1, *n* = 14; stage B2, *n* = 16), symptomatic HCM (stage C, *n* = 15), and cats with FATE (*n* = 13). Conventional and two-dimensional speckle-tracking echocardiography were performed, and LACI-ED was calculated as the ratio of LA end-diastolic volume to LV end-diastolic volume. LACI-ED increased progressively with disease severity (*p* < 0.001), showing the highest values in symptomatic HCM and FATE cases. It correlated positively with LA size and volume (*p* < 0.01) and inversely with LV GLS (*p* < 0.01). Exploratory ROC analysis for FATE status yielded limited discriminatory performance for LACI-ED > 150% (AUC = 0.575; 95% CI: 0.402–0.736; sensitivity 46.2%; specificity 84.4%). Although LACI-ED > 150% was associated with higher odds of prevalent FATE (OR = 4.65; 95% CI: 1.405–29.215; *p* = 0.020), this finding should be interpreted with caution. Pairwise comparisons of ROC curve areas between LACI-ED and conventional echocardiographic parameters (LA/Ao ratio, LA diameter, and LV GLS) revealed no statistically significant differences (all *p* > 0.05). Although LACI-ED > 150% was associated with higher odds of prevalent FATE (OR = 6.8; *p* < 0.05), this finding should be interpreted with caution. This cross-sectional study evaluates associations with disease stage and thromboembolic status at the time of examination. The findings suggest that LACI-ED reflects disease severity in feline HCM, whereas its utility for thromboembolic risk assessment appears limited.

## Simple Summary

Hypertrophic cardiomyopathy (HCM) is a common heart disease in cats and is often complicated by feline arterial thromboembolism (FATE), a major cause of morbidity and mortality. Standard echocardiographic measures usually evaluate the left atrium (LA) or left ventricle (LV) separately, potentially missing their functional interaction. The left atrioventricular coupling index (LACI) is a volumetric ratio, calculated as the LA end-diastolic volume relative to the LV end-diastolic volume, reflecting the relationship between atrial remodeling and ventricular filling. In this study of 91 cats across healthy controls, different stages of HCM, and FATE cases, LACI-ED increased progressively with disease severity. Higher LACI-ED values were associated with larger LA size and volume and with subclinical LV systolic dysfunction measured by global longitudinal strain (GLS). These results suggest that LACI-ED is associated with disease severity and prevalent thromboembolic status. Pairwise ROC comparisons demonstrated no significant differences between LACI-ED and conventional echocardiographic indices, indicating comparable rather than superior discriminatory performance. Therefore, LACI-ED may provide complementary information when interpreted alongside conventional echocardiographic indices, supporting clinical assessment and management in feline HCM.

## 1. Introduction

Feline Hypertrophic Cardiomyopathy (HCM) is the most common cardiac disease in cats and shows a variable clinical course, ranging from asymptomatic stages to CHF, arterial thromboembolism, and sudden cardiac death [1,2]. Among these complications, FATE is one of the most severe, with high morbidity and mortality, and is strongly linked to left atrial enlargement, dysfunction, and blood stasis [3,4,5].

Echocardiographic measures representing LA size (e.g., LA/Ao ratio), LA mechanical performance (including strain analysis), and left ventricular (LV) diastolic function (e.g., transmitral E/A ratio and tissue Doppler imaging) are the mainstay of current risk categorization strategies in feline HCM [6,7,8]. The integrated interplay between the LA and LV, which is crucial to the formation of elevated filling pressures, atrial remodeling, and thromboembolic risk, is not fully captured by these metrics, which primarily evaluate discrete aspects of cardiac structure and function rather than their combined relationship [9].

The left atrioventricular coupling index (LACI) has recently been introduced in human cardiology as a composite parameter reflecting the relationship between LA and LV chamber size. In most studies, LACI is preferentially assessed at end-diastole (LACI-ED), as this phase provides more reproducible and physiologically stable atrial and ventricular volumetric measurements, enabling a more robust assessment of structural remodeling through normalization of atrial size to ventricular chamber dimensions. End-systolic formulations (LACI-ES), in contrast, may be more susceptible to dynamic changes in loading conditions and ventricular contractility [7,10,11]. In human patients with HCM, increased LACI has been associated with adverse outcomes, including atrial fibrillation, stroke, and thromboembolic events, and has demonstrated independent prognostic value [12,13,14]. These findings suggest that LACI may reflect a marker of long-term LA adaptation to LV diastolic load, rather than a direct surrogate for ventricular filling properties or intrinsic atrial mechanics.

Although data in veterinary medicine are limited, atrioventricular coupling has gained increasing attention. In dogs with myxomatous mitral valve disease (MMVD), LACI has been investigated in relation to disease staging according to the American College of Veterinary Internal Medicine (ACVIM) classification, suggesting that combined atrial–ventricular parameters may provide complementary information regarding disease severity compared with isolated measurements [9]. These findings support the potential applicability of coupling-based indices such as LACI in veterinary cardiology.

From a pathophysiological perspective [10,15,16], LACI may also be associated with LV systolic performance. Left ventricular global longitudinal strain (GLS), obtained using two-dimensional speckle-tracking echocardiography, is a sensitive marker of subclinical systolic dysfunction by quantifying longitudinal myocardial fiber shortening. In HCM, longitudinal fibers, predominantly located in the subendocardium, are affected early, whereas LV ejection fraction is often preserved or even increased due to compensatory radial function [17]. Impaired LV longitudinal function may contribute to increased LV filling pressures, thereby promoting left atrial (LA) remodeling and enlargement. Accordingly, LACI may reflect the structural consequences of combined systolic and diastolic remodeling at the chamber level, rather than directly integrating functional components into a single physiological index. Although aspects of this interplay have been described in human cardiology, the underlying mechanisms remain incompletely elucidated and have not yet been investigated in veterinary species. Therefore, LACI should be interpreted primarily as a structural index reflecting chamber-level remodeling rather than a direct measure of functional coupling.

Despite these promising insights, LACI has not yet been systematically evaluated in cats with HCM, particularly in relation to disease severity and arterial thromboembolism. Therefore this retrospective cross-sectional study aimed to (i) assess the association between LACI-ED and disease severity, (ii) investigate its relationship with the presence of arterial thromboembolism at the time of examination, and (iii) explore its association with LV systolic function as assessed by global longitudinal strain. Additionally, the study aimed to evaluate the clinical relevance of LACI-ED in comparison with conventional echocardiographic parameters, including pairwise comparison of discriminatory performance using ROC curve analysis.

## 2. Materials and Methods

This retrospective, cross-sectional study was approved by the Local Ethics Committee of Bursa Uludag University (Protocol No: 2026 06/04). Client-owned cats presented to the Veterinary Teaching Hospital (Department of Internal Medicine, Faculty of Veterinary Medicine, Uludag University, Bursa, Türkiye) for cardiovascular evaluation between 2023 and 2026 were considered. A total of 120 cats were screened for eligibility. Twenty-nine cats were excluded due to restrictive cardiomyopathy (*n* = 8), non-classified cardiomyopathy (*n* = 10), or incomplete clinical data and poor image quality (*n* = 11), resulting in a final study population of 91 cats. These included 33 healthy controls, 30 asymptomatic HCM cats (14 Stage B1 and 16 Stage B2), 15 symptomatic HCM cats (Stage C), and 13 cats with FATE (Table 1). The cohort comprised multiple breeds, both sexes, and ages ranging from 1 to 14 years.

**Figure 1:**
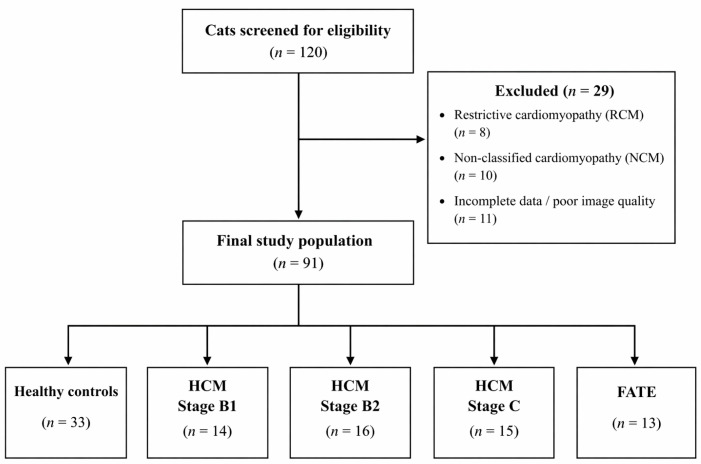
Flow diagram of case selection and study population. A total of 120 cats were initially screened. Twenty-nine cats were excluded due to restrictive cardiomyopathy (RCM, *n* = 8), non-classified cardiomyopathy (NCM, *n* = 10), or incomplete clinical data and poor image quality (*n* = 11). The final study population consisted of 91 cats, which were subsequently classified into healthy controls, HCM stages (B1, B2, and C), and cats with feline arterial thromboembolism (FATE).

A flow diagram summarizing case selection, exclusion criteria, and final group classification is provided in Figure 1.

### 2.1. HCM Diagnosis and Staging

All cats underwent physical examination, thoracic imaging, and comprehensive echocardiography prior to inclusion. Cats were classified according to ACVIM consensus criteria [3] into:

Healthy controls (Stage A): No clinical cardiac signs and normal echocardiographic parameters.

Stage B1: LV hypertrophy (IVS and/or LVFW ≥ 6 mm) with normal or mildly enlarged LA, no clinical signs of CHF, and no dynamic LVOT obstruction (peak LVOT velocity < 2.0 m/s).

Stage B2: LV hypertrophy with moderate-to-severe LA enlargement (LA/Ao ≥ 1.6, LA diameter > 15 mm, or LA volume > 2.0 mL) and evidence of atrial remodeling without overt CHF.

Stage C: Clinical signs of CHF (respiratory distress, pulmonary edema, pleural effusion) confirmed by radiography and echocardiography.

FATE: FATE was not considered an ACVIM disease stage. It was defined as an acute thromboembolic event (pain, hindlimb paresis or paralysis, weak or absent femoral pulses) occurring in cats with underlying HCM. Echocardiographic findings, such as thrombus visualization, spontaneous echo contrast, or smoke in the LA or LA appendage [3,4,5]. Cats with FATE were evaluated as a distinct group and were not included within ACVIM stage C.

### 2.2. Echocardiographic Examination

Transthoracic echocardiography was performed in unsedated cats using a Vivid S60 Ultra system (GE Healthcare, Oslo, Norway) with a 12S phased-array transducer optimized for feline imaging. Standard right parasternal short- and long-axis views and left apical views were acquired. Images were obtained during quiet respiration, with measurements averaged over ≥ 3 consecutive cardiac cycles. All images and cine loops were exported to EchoPac software (GE, version 204, Norway) for offline analysis, as previously described [18].

Conventional echocardiography included M-mode measurements of interventricular septal thickness (IVSd and IVSs), LV internal dimensions (LVIDd and LVIDs), and LV free wall thickness (LVFWd and LVFWs) at end-diastole and end-systole. Fractional shortening and ejection fraction were automatically derived using standard formulas from M-mode LV measurements [19]. LA size was quantified using the monoplane area-length method from apical four-chamber views at end-systole (maximum) and end-diastole (minimum). LA enlargement was defined by LA diameter > 15 mm, LA volume > 1 mL, or LA/Ao ratio ≥ 1.6 [20].

PW Doppler measured early (E) and late (A) diastolic transmitral flow and calculated E/A ratio. Color and CW Doppler assessed valvular regurgitation, LVOT, and aortic flow velocities, with peak systolic aortic velocity > 2.5 m/s considered consistent with dynamic LVOT [21]. Tissue Doppler imaging at the septal and lateral mitral annulus provided longitudinal myocardial velocities (S’, E’, A’). LV volumes were estimated using the Auto EF biplane module; endocardial border tracking at end-diastole was automatic, with manual tracing performed as needed [22].

For 2D-STE examination, grayscale images optimized for endocardial delineation (≥90 fps) were acquired from apical 4-chamber views over 3–5 consecutive cycles synchronized to ECG. The LV AFI module automatically traced septal, lateral, and apical segments in three layers (endocardium, myocardium, and epicardium), yielding LV GLS. Inadequate automatic tracking was corrected by manual end-systolic tracing, and persistent failures were resolved using the “reprocess” function, with ECG-guided event timing [17].

All echocardiographic measurements, including LACI-ED, were performed by a single investigator (T.V.) and subsequently reviewed and confirmed by an experienced cardiologist (Z.Y.), in order to ensure consistency and minimize observer-related variability.

### 2.3. Left Atrioventricular Coupling Index (LACI)-ED

In this study, LACI-ED was selected over LACI-ES due to its superior reproducibility and its closer association with chronic atrial remodeling relative to ventricular chamber size, a feature particularly relevant to the pathophysiology of feline HCM. LACI-ED was defined as the ratio of LA end-diastolic volume (LA-EDV) to LV end-diastolic volume (LV-EDV) during the same cardiac phase marked by mitral valve closure [9]. The apical 4-chamber long-axis view was optimized to minimize foreshortening of the LA base (Figure 1): LACI-ED = (LA-EDV/LV-EDV) × 100.

### 2.4. Inclusion and Exclusion Criteria

Healthy controls had no cardiac signs, normal hematology, IVSd and LVFWd ≤ 5 mm, LA diameter ≤ 15 mm, LA/Ao ≤ 1.5, LA volume < 2.0 mL, normal LV systolic function (LV GLS more negative than −17%, normal FS and EF), and normal diastolic function (MV E/A 1–1.5; average MV E/E’ ≤ 12) [18]. Stages B1–C and FATE cats were classified as described above.

Cats were excluded if they had secondary LV hypertrophy (systemic hypertension, hyperthyroidism), chronic kidney disease (creatinine > 1.6 mg/dL or SDMA > 14 μg/dL), anemia, dehydration, arrhythmias (atrial fibrillation, atrioventricular blocks, etc.), concurrent systemic disease, or had received cardiovascular/systemic medications within 14 days before evaluation [5,23]. Borderline wall thickness (5–6 mm) was included only with confirmatory evidence of HCM. Cats with inadequate image quality or missing stills/cine loops were excluded. Stage D HCM cats, refractory CHF requiring aggressive diuretic and maintenance therapy, were also excluded due to lack of a treatment-naive state and potential confounding effects of medications on echocardiographic parameters [3]. This exclusion was intended to minimize the influence of advanced disease and medical therapy on cardiac loading conditions and atrioventricular coupling, thereby allowing a more accurate and unbiased assessment of LACI-ED.

### 2.5. Statistics

All statistical analyses were performed using SigmaPlot Statistical and Graphing Software (Version 12.5, CA, USA) and MedCalc (version 20.218, Ostend, Belgium). Continuous variables are presented as mean ± standard deviation (SD), with ranges reported where appropriate. Normality of data distribution was assessed using the Shapiro–Wilk test, and homogeneity of variances was evaluated using Levene’s test. Comparisons among groups (A, B1, B2, and C) were performed using one-way analysis of variance (ANOVA) with Tukey’s post hoc tests when assumptions of normality and equal variance were met; otherwise, the Kruskal–Wallis test with Dunn’s post hoc test was applied. Based on Shapiro–Wilk testing, variables such as LACI-ED and left atrial volume showed non-normal distribution, whereas LV GLS was normally distributed (see Table 1 for full details).

LV GLS was analyzed using signed values and expressed as negative percentages, consistent with standard speckle-tracking echocardiography convention. To assess the discriminatory performance of selected parameters (LV GLS and LACI-ED) for asymptomatic versus symptomatic HCM, cats in stages B1 and B2 were pooled and compared with Stage C cats and healthy controls. A similar comparison was performed between the overall HCM cohort (stages B1, B2, and C combined) and cats with FATE [18].

Correlations between LACI-ED and clinical or echocardiographic variables were assessed using Spearman correlation. Given the exploratory nature of these analyses, no formal adjustment for multiple comparisons was applied; therefore, results were interpreted cautiously in the context of biological plausibility and consistency across variables rather than isolated *p*-values.

Multivariable linear regression analysis was performed with LACI-ED as the dependent variable. An enter (simultaneous inclusion) model was used, and variables were selected a priori based on clinical relevance and established associations with cardiac remodeling in feline HCM. Age and body weight were included a priori as clinical covariates due to their potential confounding effects. To minimize redundancy, only one representative variable from each collinear parameter group was included. Multicollinearity was assessed using the variance inflation factor (VIF), and all values were <2.0, indicating no relevant multicollinearity among predictors (Supplementary Table S2).

Receiver operating characteristic (ROC) curve analysis was conducted to evaluate the discriminatory performance of LACI-ED between HCM and FATE, including estimation of the area under the curve (AUC) with 95% confidence intervals (CI), sensitivity, and specificity. The optimal cut-off value was determined using the Youden index; however, given the modest discriminatory performance, these cut-off values should be considered exploratory. In addition, pairwise comparisons of ROC curve areas between LACI-ED and conventional echocardiographic parameters (LA/Ao ratio, LA diameter, and LV GLS) were performed using the DeLong method for correlated ROC curves.

Based on the ROC-derived optimal LACI-ED cut-off values, odds ratios (OR) with 95% CI were calculated to estimate the risk of FATE in cats with symptomatic HCM, and chi-square tests were used for group comparisons. For non-normally distributed variables, data are presented as median and interquartile range (IQR), together with distributional indices such as skewness and kurtosis. Intra- and inter-observer reproducibility of LACI-ED measurements was assessed using the intraclass correlation coefficient (ICC) in 10 randomly selected cats. A *p*-value < 0.05 was considered statistically significant. All figures were generated using SigmaPlot Statistical and Graphing Software (Version 12.5, CA, USA).

## 3. Results

Descriptive statistics are presented in Table 1 as mean ± SD for all variables to facilitate comparison across groups. For variables with non-normal distribution, including LACI-ED and LA volume, additional reporting using median (IQR; Q1–Q3) is provided in the text. Detailed distributional indices, including mean, standard error of the mean, median, IQR, skewness, kurtosis, and Shapiro–Wilk test results, are presented in Supplementary Table S1.

### 3.1. Study Population

Age and body weight did not differ among groups (*p* = 0.495 and *p* = 0.078, respectively). Heart rate was higher in HCM C compared with controls (*p* < 0.05), whereas no difference was observed between FATE and controls (*p* = 0.914) (Table 1).

### 3.2. Echocardiographic Findings

Interventricular septal thickness (IVSd and IVSs) and left ventricular free wall thickness (LVFWd and LVFWs) were increased in HCM groups compared with controls (Table 1). IVSd was significantly higher in stages B1 and C (both *p* < 0.001), whereas no significant difference was observed in stage B2 (*p* = 0.076). IVSs was significantly increased in stages B1 and B2 (both *p* < 0.01) and in stage C (*p* < 0.001). LVFWd was significantly increased in stages B1, B2, and C (all *p* < 0.001). LVFWs was significantly higher in stage B1 (*p* < 0.001), stage B2 (*p* < 0.01), and stage C (*p* < 0.001). The highest wall thickness values were observed in stage C.

Left atrial size parameters increased progressively with disease severity. LA/Ao was significantly higher in stages B2 and C (both *p* < 0.001). In addition, LA/Ao differed significantly between B2 and B1 (*p* < 0.01) and between C and B1 (*p* < 0.001). LA diameter was significantly increased in all HCM groups compared with controls (all *p* < 0.001). LA volume was significantly elevated in stage C compared with controls (*p* < 0.001) and stage B1 (*p* < 0.001), whereas no significant differences were observed in earlier stages (A vs. B1, *p* = 0.915; A vs. B2, *p* = 0.087; B1 vs. B2, *p* = 0.208). In contrast, LV internal dimensions (LVIDd and LVIDs), FS, and transmitral E/A ratio did not differ among groups (Table 1).

**Figure 2:**
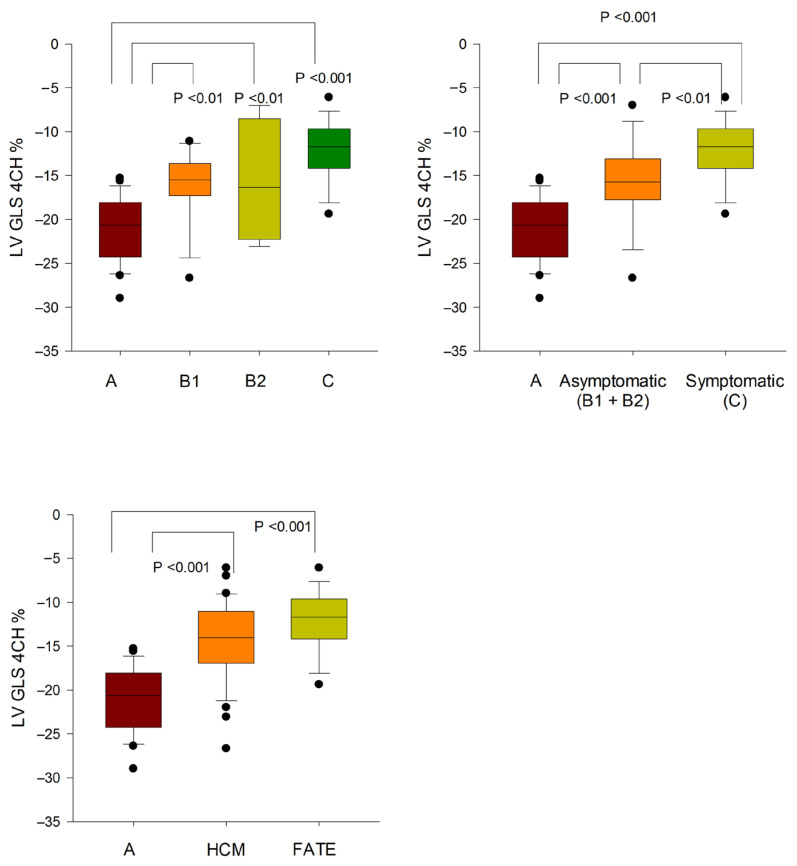
Left ventricular global longitudinal strain (LV GLS) obtained from the apical four-chamber (4Ch) view in cats across stages of hypertrophic cardiomyopathy (HCM) and in cats with feline arterial thromboembolism (FATE). Data are presented as median (IQR; Q1–Q3). Boxes indicate interquartile ranges, central lines represent medians, and whiskers denote minimum–maximum values. Statistically significant differences between groups are indicated (*p* < 0.05–0.001). LV GLS decreased progressively with increasing disease severity, with the lowest values observed in symptomatic HCM and FATE groups, indicating impaired systolic function. More negative values indicate better systolic myocardial deformation, whereas values closer to zero reflect reduced systolic function.

LV GLS was significantly reduced in all HCM groups compared with controls (B1, *p* < 0.01; B2, *p* < 0.05; C, *p* < 0.001). No significant differences were detected among HCM stages (B1 vs. B2, *p* = 0.840; B1 vs. C, *p* = 0.057; B2 vs. C, *p* = 0.168), although a trend toward lower values was observed in stage C compared with B1. When stratified by clinical status, LV GLS was lower in both asymptomatic (−15.9 ± 4.8%) and symptomatic cats (−12.2 ± 3.3%) than in controls (both *p* < 0.001), with a further reduction in symptomatic compared with asymptomatic HCM (*p* < 0.01). LV GLS was also decreased in cats with FATE compared with controls (*p* < 0.001), whereas no significant differences were observed between FATE and HCM cats (*p* = 0.115), or between FATE and stage C HCM (*p* = 1.000) (Figure 2).

### 3.3. LACI-ED

**Figure 3:**
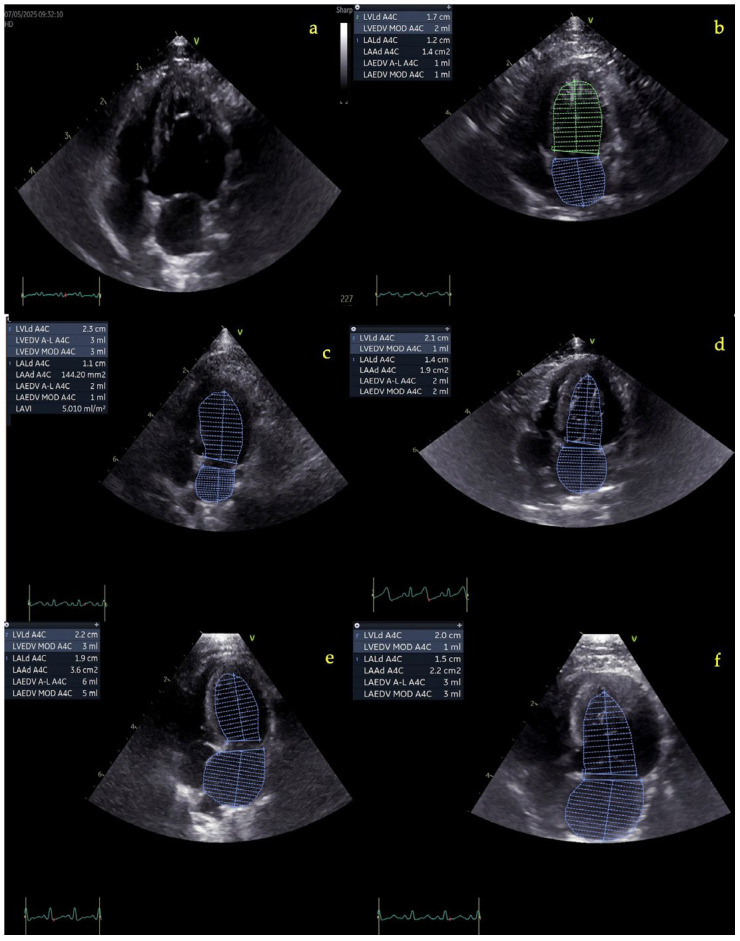
Left atrioventricular coupling index at end-diastole (LACI-ED) assessment across HCM stages and FATE. LACI was calculated as the ratio of left atrial (LA) volume to left ventricular (LV) volume at end-diastole. Echocardiographic images were acquired from the left apical four-chamber view with ECG gating. Endocardial borders of the LA and LV were manually traced to determine chamber volumes. Representative images are shown for: (**a**) a healthy control cat before measurement, (**b**) healthy control after tracing (LACI: 50%), (**c**) HCM stage B1 (66.6%), (**d**) HCM stage B2 (100%), (**e**) HCM stage C (200%), and (**f**) feline arterial thromboembolism (FATE) (300%). LACI-ED increases progressively from healthy cats to symptomatic HCM and FATE, reflecting progressive left atrial enlargement relative to left ventricular size.

**Figure 4:**
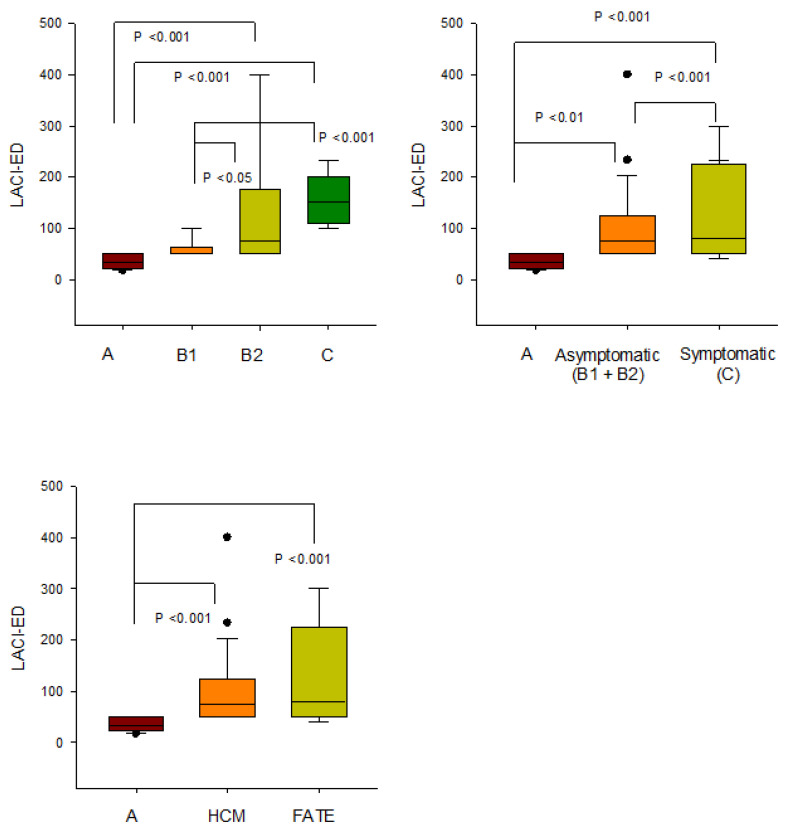
Distribution of the left atrioventricular coupling index at end-diastole (LACI-ED) in healthy cats (Stage A), across hypertrophic cardiomyopathy (HCM) stages (B1, B2, and C), and in cats with feline arterial thromboembolism (FATE). Data are presented as median (IQR; Q1–Q3). Boxes indicate interquartile ranges, central lines represent medians, and whiskers denote minimum–maximum values. Statistically significant differences between groups are indicated (*p* < 0.05–0.001). LACI-ED increased progressively across disease stages, with the highest values observed in advanced (stage C) HCM and FATE groups, consistent with progressive atrial and ventricular structural remodeling.

Representative LACI-ED measurements from selected cases with varying HCM severity and FATE are illustrated in Figure 3. Group differences in LACI-ED expressed as mean ± SD are shown in Figure 4, whereas median (IQR; Q1–Q3) values and detailed distributional statistics are provided in Supplementary Table S1.

LACI-ED increased significantly across disease stages. Median (IQR; Q1–Q3) values were 33.3 (22.3–50.0) in healthy controls, 50.0 (50.0–62.5) in stage B1, 75.0 (50.0–175.0) in stage B2, and 150.0 (108.3–200.0) in stage C. Compared with controls, LACI-ED was significantly higher in stages B2 (*p* < 0.001) and C (*p* < 0.001), whereas no significant difference was observed in stage B1 (*p* = 0.181). A significant increase was also observed in stage B2 compared with stage B1 (*p* < 0.05), while no difference was found between stages B2 and C (*p* = 0.193).

When stratified by clinical status, both asymptomatic (B1 + B2) and symptomatic (Stage C) cats exhibited significantly higher LACI-ED values compared with controls (*p* < 0.01 and *p* < 0.001, respectively). In addition, LACI-ED was significantly higher in symptomatic compared with asymptomatic HCM (*p* < 0.001), with median values of 50.0 (50.0–100.0) and 150.0 (108.3–200.0), respectively.

Cats with FATE had a median LACI-ED of 80.0 (50.0–225.0), which was significantly higher than controls (*p* < 0.001), but did not differ significantly from the overall HCM cohort (*p* = 0.191). Overall, pooled HCM cats (stages B1, B2, and C combined) demonstrated significantly higher LACI-ED values than controls [75.0 (50.0–124.8) vs. 33.3 (22.3–50.0); *p* < 0.001] (Figure 3 and Figure 4).

### 3.4. Correlations and Risk Estimations

No adjustment for multiple comparisons was applied, as analyses were exploratory. Indices of LA size and remodeling, such as LA volume (*r* = 0.502, *p* = 0.0003), LA diameter (*r* = 0.422, *p* = 0.0029), and LA/Ao ratio (*r* = 0.322, *p* = 0.025), were strongly correlated with LACI-ED, according to Spearman correlation analysis. Additionally, LACI-ED showed a moderate positive correlation with LVPWd (*r* = 0.399, *p* = 0.0051). Furthermore, there was a negative correlation between LAC-ED and GLS (*r* = −0.361, *p* = 0.012). Heart rate (*r* = −0.209, *p* = 0.333) and age (*r* = 0.206, *p* = 0.164) did not significantly correlate with LACI-ED. Body weight and LACI-ED had a weak but statistically significant positive connection (*r* = 0.303, *p* = 0.036).

**Figure 5:**
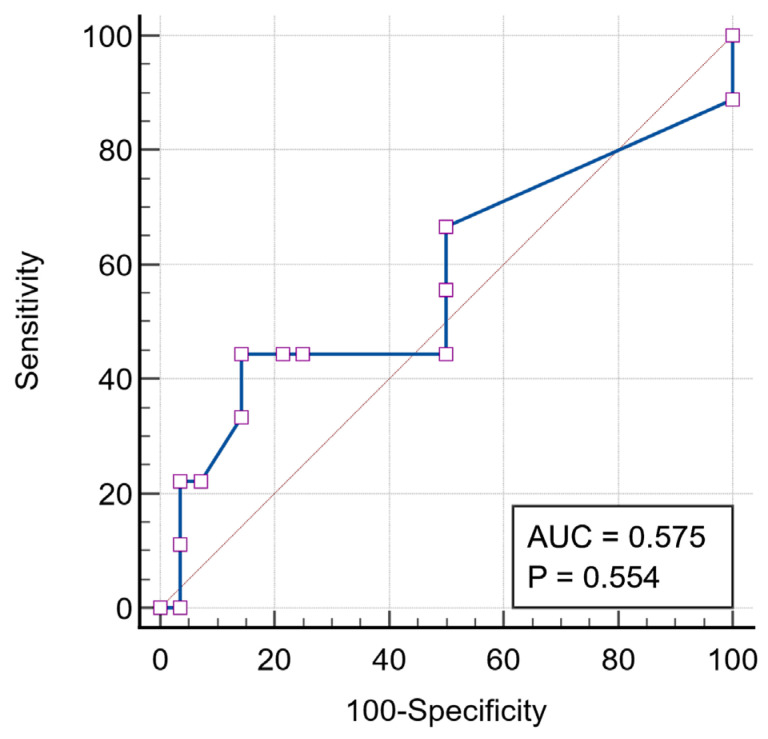
Exploratory ROC analysis of LACI-ED for discriminating cats with FATE from comparator cats. The optimal Youden-derived threshold was >150%, but overall discriminatory performance was limited. The blue line represents the ROC curve, while the diagonal reference line indicates no discriminative ability (AUC = 0.5).

In multiple linear regression analysis, the overall model was not statistically significant (r^2^ = 0.281, *p* = 0.108), and LACI-ED was not independently associated with any of the examined clinical (age, body weight, and heart rate) and echocardiographic variables (LA diameter, LA volume, LV FS, and LV GLS 4CH). No statistically significant independent associations were identified between LACI-ED and the evaluated variables. Detailed regression outputs are provided in Supplementary Table S2. For thromboembolic status, a cut-off value of LACI-ED >150% yielded an AUC of 0.575 (95% CI: 0.402–0.736), with a sensitivity of 46.2% and specificity of 84.4% (Figure 5). Using this threshold, 6/13 cats with FATE and 7/45 non-FATE HCM cats were classified above the cut-off, whereas 7/13 FATE cats and 38/45 non-FATE HCM cats remained below the threshold (Supplementary Table S3). LACI-ED > 150% was associated with increased odds of FATE (OR = 4.65; 95% CI: 1.405–29.215; *p* = 0.020). Pairwise comparison of ROC curve areas between LACI-ED and conventional echocardiographic parameters (LA/Ao ratio, LA diameter, and LV GLS) revealed no statistically significant differences (all *p* > 0.05). (Table 2) (Supplementary Table S3).

Reproducibility analysis demonstrated excellent agreement for LACI-ED measurements. Intra-observer reliability was high, with an intraclass correlation coefficient (ICC) of 0.962 (95% confidence interval [CI]: 0.890–0.993). Inter-observer agreement was also excellent, with an ICC of 0.954 (95% CI: 0.896–0.981).

## 4. Discussion

The present study suggests that LACI-ED is closely associated with both disease severity and the presence of thromboembolic events in cats with HCM. By relating LA remodeling to LV chamber geometry, LACI-ED serves as a structural surrogate of the long-term left atrial response to left ventricular diastolic load, rather than a dynamic measure of cardiac functional coupling. To the authors’ knowledge, this is the first systematic evaluation of LACI-ED in feline HCM and its association with FATE. Conventional parameters such as LA/Ao ratio and LA volume primarily reflect structural remodeling, while indices of atrial function and GLS assess isolated aspects of atrial or ventricular performance. In contrast, LACI-ED integrates atrial size and ventricular geometry into a single parameter, providing a composite structural assessment of cardiac remodeling; however, ROC curve comparisons in the present study demonstrated no statistically significant differences between LACI-ED and conventional echocardiographic parameters, suggesting broadly comparable discriminatory performance rather than clear superiority. This integrative approach may be particularly useful in cases where conventional parameters are inconclusive or discordant, although its incremental diagnostic value over established indices was not demonstrated in the present analysis [9,15,24]. Importantly, LACI should be interpreted as a structural surrogate reflecting the relationship between LA remodeling and LV chamber size at end-diastole, and not as a direct measure of ventricular filling dynamics or intrinsic atrial mechanics.

Left ventricular hypertrophy in cats has been previously shown to promote LA enlargement and early atrial dysfunction [1,3,7]. In early-stage (B1) HCM, subtle increases in LV filling pressures can induce initial LA dilation and mild atrial dysfunction, reflected modestly by LACI-ED elevation. Moderate-stage (B2) disease demonstrates further LA dilation and diastolic impairment, producing higher LACI values consistent with a rising hemodynamic burden. Symptomatic cats (Stage C) are characterized by marked atrial remodeling, consistent with advanced structural adaptation and impaired ventricular compliance. These observations are in agreement with ACVIM guidelines, which emphasize that progression from subclinical to overt disease is accompanied by worsening atrial remodeling and diastolic filling abnormalities [3].

The observed negative correlation between LACI-ED and LV GLS suggests a potential relationship between ventricular longitudinal function, combined atrial–ventricular remodeling in HCM. GLS is widely regarded as a sensitive marker of subclinical systolic dysfunction, particularly when conventional indices such as ejection fraction remain preserved [17,23]. In the present study, less negative LV GLS values in symptomatic HCM may reflect impaired myocardial deformation. The underlying mechanisms linking these findings cannot be directly determined from our data. However, based on previous studies, impaired longitudinal deformation in HCM has been associated with myocardial disarray, fibrosis, and microvascular dysfunction, which may contribute to altered ventricular relaxation and increased filling pressures [1,25,26]. These changes may, in turn, be associated with progressive atrial remodeling. Within this context, LACI provides a combined volumetric description of LA and LV geometry and may reflect the combined structural alteration involving both chambers [24]. Importantly, the absence of independent associations in multivariable analysis suggests that LACI-ED may share variance with established LA size parameters and related geometric indices, rather than representing an independent functional marker. Nevertheless, these mechanistic interpretations should be considered hypothesis-generating, as no histological, biomarker, or invasive hemodynamic measurements were available in the present study to directly support these associations. In addition, the multivariable regression model, constructed a priori with assessment of multicollinearity (all VIF < 2) and without stepwise selection, supports the robustness of these findings by minimizing model instability and redundancy. Consistent with these findings, ROC-based comparisons further indicated that LACI-ED did not significantly outperform GLS or conventional LA size parameters, reinforcing the interpretation that these indices may reflect overlapping aspects of cardiac remodeling rather than distinct diagnostic domains.

Human studies provide further support for the relevance of LACI as a structural imaging-derived parameter. Elevated LACI is associated with advanced diastolic dysfunction, increased LA volumes, and higher risks of atrial fibrillation and heart failure events, with thresholds of approximately 33–36% identifying patients at elevated risk [7,8,27,28]. However, these thresholds are substantially lower than those observed in the present study. This discrepancy likely reflects species-specific differences in cardiac geometry and chamber size, as well as methodological variations in LACI calculation, including the timing of LA volume measurement (e.g., end-diastolic vs. maximal or minimal volumes) and the lack of body size indexation. Therefore, direct comparison between human and feline LACI values should be made with caution, and human-derived thresholds cannot be considered directly applicable to cats.

In feline HCM, progressive LA enlargement represents a central structural abnormality closely linked to disease severity and thromboembolic risk [7,28]. In this context, LACI-ED may reflect the relationship between LA remodeling and LV filling-related geometry, capturing the extent of structural remodeling across both chambers beyond conventional echocardiographic parameters [7,8,13]. Nevertheless, veterinary evidence remains limited, and further prospective studies are required to define clinically relevant cut-off values for cats with HCM.

In this cohort, cats with FATE had higher LACI-ED values compared to controls and non-thromboembolic HCM cats. LACI-ED >150% was associated with the presence of FATE, albeit with modest overall discriminatory ability (AUC 0.575) and wide confidence intervals. Importantly, these findings reflect cross-sectional associations rather than predictive performance. The use of a data-derived cut-off within the same dataset may introduce optimism bias and overestimate discriminative ability. Therefore, LACI-ED should be interpreted as a supportive rather than a standalone marker. LA enlargement and dysfunction are established contributors to thrombus formation in feline HCM, mediated by blood stasis, spontaneous echo contrast, and hypercoagulability [2,4,18]. Conventional echocardiographic measures, such as LA/Ao ratio and LA diameter, provide static assessment of chamber size but do not capture the dynamic interaction between atrial volume and ventricular filling. By integrating atrial size and ventricular geometry into a single structural index, LACI may reflect the chronic remodeling patterns observed in cats with FATE. Direct comparisons with established echocardiographic markers (LA/Ao ratio, LA diameter, and GLS) using ROC curve analysis revealed no statistically significant differences in discriminatory performance, indicating that LACI-ED does not provide incremental discrimination beyond conventional indices within this dataset. These findings suggest that the clinical relevance of LACI-ED may lie more in its integrative structural interpretation rather than improved diagnostic accuracy compared to individual conventional parameters.

Translationally, LACI has been investigated across species as a structural imaging-derived parameter associated with adverse cardiac conditions. In humans, it predicts adverse outcomes in chronic coronary syndrome, heart failure, and HCM with preserved ejection fraction, demonstrating association with cardiac conditions in addition to conventional parameters [10,14]. In dogs with myxomatous mitral valve disease, atrioventricular coupling indices improve discrimination of disease severity relative to isolated measurements [29]. Despite pathophysiological differences between canine valve disease [30] and feline HCM [3], both conditions involve atrial remodeling and altered filling dynamics, reinforcing the relevance of structural remodeling–based indices for comprehensive cardiac assessment.

From a clinical perspective, LACI-ED can be readily obtained from routine echocardiographic examinations without requiring additional imaging techniques or specialized software. This makes it a practical and time-efficient parameter that may complement routine clinical assessment, although its added diagnostic value relative to conventional measurements appears limited based on the present findings.

This study’s retrospective, cross-sectional design limits causal inference and precludes longitudinal evaluation of LACI-ED relative to disease progression or FATE development. The retrospective nature of the study may also introduce selection bias as inclusion depended on the availability of complete clinical and echocardiographic data. Furthermore, the absence of longitudinal follow-up prevents assessment of temporal changes in LACI-ED and limits evaluation of its prognostic significance. Although ROC curve comparisons were performed, the sample size may have limited the statistical power to detect small but clinically relevant differences between LACI-ED and conventional echocardiographic parameters. Additionally, two-dimensional echocardiographic assessment of LACI-ED relies on geometric assumptions, which may introduce measurement variability, although image optimization and cycle averaging were applied to mitigate this. Finally, exclusion of stage D HCM cats (refractory CHF) limits generalizability to treatment-naive cats and advanced-stage populations.

However, despite these limitations, LACI-ED measurements demonstrated excellent reproducibility, with intraclass correlation coefficients (ICC) of 0.962 for intra-observer and 0.954 for inter-observer agreement, supporting its robustness as a quantitative echocardiographic parameter.

Future studies should include a prospective, longitudinal design with larger and more balanced cohorts to validate the prognostic utility of LACI-ED, define clinically relevant cut-off values, and further clarify its role in thromboembolic risk status in feline HCM.

## 5. Conclusions

In conclusion, LACI-ED is associated with disease severity, myocardial dysfunction, and prevalent thromboembolic status in feline HCM. As a volumetric ratio integrating LA and LV volumes at end-diastole, it provides a composite structural index of atrial and ventricular remodeling beyond isolated chamber measurements, although no significant superiority over conventional echocardiographic parameters was demonstrated in ROC-based comparisons. Elevated LACI-ED values are observed more frequently in cats with more advanced disease and in those with prevalent thromboembolic status; however, its discriminatory performance remains modest, and its clinical interpretation should therefore be made with caution. These findings warrant further validation in prospective studies to define clinically relevant thresholds and clarify their clinical relevance in feline HCM.

## Figures and Tables

**Table 1:** Selected clinical and echocardiographic parameters in cats across stages of hypertrophic cardiomyopathy (HCM), and in cats with feline arterial thromboembolism (FATE). Mean ± SD (minimum–maximum).

Parameters	A (Healthy) *n* = 33	HCM B1 *n* = 14	HCM B2 *n* = 16	HCM C *n* = 15	FATE *n* = 13
Age (years) ^α^	4.2 ± 3.0 ^a^ (1.0–14.0)	5.9 ± 3.2 ^a^ (1.0–11.0)	4.1 ± 3.7 ^a^ (1.0–12.0)	5.3 ± 3.8 ^a^ (1.0–13.0)	5.0 ± 2.1 ^a^ (2.0–8.0)
BW (kg) ^β^	4.1 ± 1.0 ^a^ (2.1–6.0)	4.8 ± 0.6 ^a^ (3.5–6.0)	3.6 ± 0.6 ^a^ (3.0–4.5)	4.1 ± 0.7 ^a^ (2.7–5.2)	4.0 ± 0.9 ^a^ (3.0–6.2)
HR (bpm) ^β^	183 ± 21 ^a^ (133–224)	186 ± 28 ^ab^ (121–219)	212 ± 36 ^ab^ (173–270)	214 ± 40 ^b*^ (127–269)	182 ± 25 ^a^ (144–234)
IVSd mm ^β^	4.1 ± 0.6 ^a^ (2.6–5.0)	5.9 ± 1.3 ^b***^ (3.3–7.8)	5.6 ± 2.0 ^ab^ (3.0–8.5)	6.4 ± 1.9 ^b***^ (3.5–10.0)	6.7 ± 1.4 ^#***^ (5.1–10.2)
IVSs mm ^β^	6.0 ± 0.8 ^a^ (4.1–7.7)	7.7 ± 1.5 ^b**^ (4.3–10.6)	8.0 ± 1.8 ^b**^ (5.8–10.4)	8.7 ± 2.0 ^b***^ (4.8–11.9)	8.0 ± 1.4 ^#***^ (6.2–10.9)
LVIDd mm ^β^	13.9 ± 2.0 ^a^ (10.2–18.1)	13.7 ± 2.1 ^a^ (9.0–17.2)	12.4 ± 2.5 ^a^ (8.8–15.3)	13.4 ± 2.1 ^a^ (9.3–15.9)	12.6 ± 2.6 (7.6–16.1)
LVIDs mm ^β^	7.7 ± 1.5 ^a^ (4.5–10.6)	7.4 ± 1.9 ^a^ (4.5–11.2)	6.8 ± 2.3 ^a^ (3.7– 9.7)	7.2 ± 2.0 ^a^ (3.2–10.0)	7.8 ± 2.1 (4.2–11.1)
LVFWd mm ^α^	4.5 ± 0.7 ^a^ (3.4–6.0)	6.6 ± 1.3 ^b***^ (4.6–9.3)	7.7 ± 1.1 ^b***^ (6.5–9.7)	7.3 ± 1.2 ^b***^ (3.7–8.9)	7.6 ± 2.1 ^#***^ (4.5–11.2)
LVFWs mm ^β^	6.7 ± 1.1 ^a^ (5.0–9.8)	8.8 ± 1.6 ^b***^ (5.0–11.4)	8.9 ± 0.9 ^b**^ (8.1–10.8)	9.1 ± 1.2 ^b***^ (6.9–11.4)	9.0 ± 1.8 ^#***^ (6.7–13.3)
LA/Ao ^α^	1.2 ± 0.1 ^a^ (1.0–1.5)	1.3 ± 0.1 ^a^ (1.1–1.5)	1.7 ± 0.2 ^b***∏**^ (1.6–2.1)	1.8 ± 0.4 ^b***∏***^ (1.2–2.8)	1.8 ± 0.3 ^#***∏***^ (1.3–2.3)
LAd mm ^α^	5.3 ± 6.0 ^a^ (0.7–15.0)	11.6 ± 1.5 ^b***^ (9.0–15.0)	13.0 ± 1.0 ^b***^ (11.0–14.0)	15.4 ± 3.4 ^b***^ 12.0–24.0)	17.4 ± 3.4 ^#***∏**^ (12.2–24.0)
LAV mL ^α^	1.0 ± 0.2 ^a^ (0.7–2.0)	1.2 ± 0.4 ^a^ (1.0–2.0)	2.6 ± 1.2 ^ab^ (1.0–4.0)	4.1 ± 2.5 ^b***∏***^ (2.0–11.0)	4.1 ± 2.1 ^#***∏***^ (1.0–7.0)
FS% ^β^	44.5 ± 6.7 ^a^ (34.0–59.1)	45.5 ± 11.5 ^a^ (19.1–65.0)	46.8 ± 10.7 ^a^ (32.0–62.4)	45.6 ± 13.6 ^a^ (26.3–74.0)	38.5 ± 11.2 ^a^ (18.4–55.1)
Mitral E/A m/s ^β^	1.2 ± 0.2 ^a^ (0.7–1.7)	1.1 ± 0.2 ^a^ (0.9–1.5)	1.2 ± 0.0 ^a^ (1.1–1.3)	1.1 ± 0.6 ^a^ (0.6–2.7)	1.2 ± 0.4 ^a^ (0.8–2.0)

^α^ represents variables with normal distribution (*p* > 0.05 in Shapiro–Wilk test), while ^β^ represents variables with non-normal distribution (*p* < 0.05). ^a, b^: Values within the same row sharing the same letter are not significantly different. ^∏^ compared to HCM B1, ^#^ compared to healthy controls * *p* < 0.05, ** *p* < 0.01, and *** *p* < 0.001.

**Table 2:** Pairwise comparison of ROC curve areas for LACI-ED and conventional echocardiographic parameters in the classification of FATE.

Comparison	ΔAUC	SE	95% CI	*z*	*p*-Value
LACI-ED vs. LA/Ao ratio	0.147	0.236	−0.315 to 0.608	0.623	0.533
LACI-ED vs. LA diameter	0.283	0.228	−0.164 to 0.730	1.243	0.214
LACI-ED vs. GLS	0.180	0.132	−0.078 to 0.438	1.368	0.171
LA/Ao ratio vs. LA diameter	0.137	0.099	−0.058 to 0.332	1.375	0.169
LA/Ao ratio vs. GLS	0.033	0.171	−0.301 to 0.368	0.195	0.845
LA diameter vs. GLS	0.103	0.199	−0.287 to 0.493	0.519	0.604

ΔAUC: difference between areas under the curve; SE: standard error; CI: confidence interval. No statistically significant differences were observed between parameters (all *p* > 0.05).

## Data Availability

The raw data supporting the conclusions of this article will be made available by the authors on request.

## Supplementary Materials

The following supporting information can be downloaded at: https://www.mdpi.com/article/10.3390/vetsci13050491/s1, Supplementary Materials: Table S1. Selected clinical and echocardiographic parameters in cats across stages (B1, B2, and C) of hypertrophic cardiomyopathy (HCM), and in cats with feline arterial thromboembolism (FATE). Table S2. Multiple linear regression analysis of determinants of LACI-ED. Table S3. Contingency table underlying ROC-derived classification of FATE using the LACI-ED >150% threshold.

## Abbreviations

The following abbreviations are used in this manuscript:

2D-STE	Two-Dimensional Speckle-Tracking Echocardiography
ACVIM	American College of Veterinary Internal Medicine
AFI	Automated Function Imaging
AUC	Area Under the Curve
CHF	Congestive Heart Failure
CI	Confidence Interval
CW (Doppler)	Continuous-Wave Doppler
E/A Ratio	Early-to-Late Ventricular Filling Velocity Ratio
EF	Ejection Fraction
FATE	Feline Arterial Thromboembolism
FS	Fractional Shortening
GLS	Global Longitudinal Strain
HCM	Hypertrophic Cardiomyopathy
IVS	Interventricular Septum
LA	Left Atrium
LA/Ao	Left Atrium to Aorta Ratio
LACI	Left Atrioventricular Coupling Index
LACI-ED	Left Atrioventricular Coupling Index at End-Diastole
LA-EDV	Left Atrial End-Diastolic Volume
LV	Left Ventricle
LV-EDV	Left Ventricular End-Diastolic Volume
LVFW	Left Ventricular Free Wall
LVOT	Left Ventricular Outflow Tract
OR	Odds Ratio
PW (Doppler)	Pulsed-Wave Doppler
ROC	Receiver Operating Characteristic
SDMA	Symmetric Dimethylarginine
TDI	Tissue Doppler Imaging
VIF	Variance Inflation Factor
