# Supplementary material for: Left Atrioventricular Coupling Index in Feline Hypertrophic Cardiomyopathy: Association with Disease Severity and Arterial Thromboembolism
Source: Vet Sci. 2026 May 19;13(5):491. doi: 10.3390/vetsci13050491 (PMC13211497; doi:10.3390/vetsci13050491)
Supplement: Supplementary file 1 [file vetsci-13-00491-s001.zip › vetsci-4279526-supplementary.pdf]

Supplementary Table S1. Selected clinical and echocardiographic parameters in cats across stages (B1, B2, and C) of hypertrophic cardiomyopathy (HCM), and in cats with feline arterial thromboembolism (FATE).

Parameters	n	Missing	Mean	SE	Median	SD	Min	Max	IQR Q1	IQR Q3	Skewness	Kurtosis	SWilkProb
**Age (yrs)**													
Healthy	33	0	4,223	0,559	4,000	3,061	1,000	14,000	1,500	6,000	1,153	1,951	0,003
B1	14	0	5,929	0,874	6,000	3,269	1,000	11,000	3,500	9,000	-0,126	-1,076	0,594
B2	16	0	4,125	1,329	2,500	3,758	1,000	12,000	1,250	6,000	1,507	2,152	0,045
C	15	0	5,308	1,058	3,000	3,816	1,000	13,000	3,000	9,500	0,943	-0,505	0,018
FATE	13	0	5,000	0,647	5,000	2,145	2,000	8,000	3,000	8,000	0,446	-1,065	0,103
**BW (kg)**													
Healthy	33	0	4,161	0,172	4,300	1,058	2,100	6,000	3,475	5,000	-0,351	-0,599	0,211
B1	14	0	4,821	0,171	4,800	0,641	3,500	6,000	4,500	5,200	-0,173	0,544	0,926
B2	16	0	3,686	0,240	3,500	0,636	3,000	4,500	3,000	4,300	0,180	-2,217	0,195
C	15	0	4,190	0,248	4,500	0,785	2,700	5,200	3,500	4,775	-0,643	-0,337	0,442
FATE	13	0	4,050	0,284	3,950	0,984	3,000	6,200	3,075	4,600	0,815	0,417	0,190
**HR (bpm)**													
Healthy	33	0	183,920	4,343	184,000	21,714	133,00	224,00	173,000	200,500	-0,504	0,215	0,726
B1	14	0	186,571	7,723	195,500	28,897	121,00	219,00	163,500	209,250	-0,923	0,384	0,193
B2	16	0	212,375	12,869	207,500	36,398	173,00	270,00	182,750	250,500	0,865	-0,612	0,119
C	15	0	214,214	10,951	221,500	40,973	127,00	269,00	194,500	244,250	-0,974	0,584	0,173
FATE	13	0	182,750	7,357	181,000	25,485	144,00	234,00	165,250	201,000	0,503	0,0680	0,984
**IVSd mm**													
Healthy	33	0	4,186	0,126	4,125	0,689	2,660	5,090	3,573	4,830	-0,371	-0,977	0,042
B1	14	0	5,999	0,372	5,960	1,391	3,340	7,840	5,412	7,058	-0,764	0,0213	0,185
B2	16	0	5,680	0,766	6,210	2,026	3,030	8,570	4,030	7,530	0,108	-1,397	0,666
C	15	0	6,494	0,508	6,165	1,903	3,530	10,090	5,127	8,380	0,383	-0,661	0,840
FATE	13	0	6,725	0,427	6,195	1,479	5,170	10,240	5,598	7,565	1,401	1,766	0,062
**IVSs mm**													
Healthy	33	0	6,075	0,164	6,095	0,899	4,110	7,750	5,690	6,710	-0,347	-0,0336	0,536
B1	14	0	7,703	0,423	7,590	1,582	4,340	10,690	7,172	8,725	-0,264	1,014	0,562
B2	16	0	8,044	0,694	8,070	1,836	5,820	10,470	5,820	10,140	0,0551	-1,175	0,292
C	15	0	8,741	0,557	8,960	2,084	4,880	11,980	7,713	10,080	-0,540	-0,163	0,562

FATE	13	0	8,085	0,425	8,025	1,471	6,280	10,970	6,740	8,555	0,731	0,256	0,184
**LVIDd mm**													
Healthy	33	0	13,902	0,370	14,360	2,025	10,240	18,100	11,990	15,085	-0,00503	-0,652	0,437
B1	14	0	13,724	0,581	13,405	2,175	9,080	17,260	12,385	15,758	-0,168	0,341	0,466
B2	16	0	12,411	0,954	13,030	2,524	8,830	15,360	9,900	14,590	-0,331	-1,756	0,507
C	15	0	13,479	0,562	14,240	2,101	9,390	15,930	12,048	15,198	-0,608	-0,733	0,187
FATE	13	0	12,632	0,751	13,075	2,602	7,660	16,140	10,948	14,860	-0,473	-0,675	0,574
**LVIDs mm**													
Healthy	33	0	7,751	0,287	7,805	1,570	4,580	10,600	7,120	8,623	-0,317	-0,0497	0,238
B1	14	0	7,470	0,523	7,215	1,958	4,580	11,210	5,730	8,665	0,413	-0,321	0,797
B2	16	0	6,811	0,897	5,970	2,373	3,720	9,780	4,970	9,700	0,236	-1,647	0,415
C	15	0	7,294	0,540	7,450	2,021	3,280	10,050	5,880	9,240	-0,388	-0,389	0,728
FATE	13	0	7,823	0,619	8,215	2,144	4,240	11,170	5,652	9,515	-0,205	-0,981	0,855
**LVFWd mm**													
Healthy	33	0	4,592	0,144	4,620	0,787	3,400	6,000	3,933	5,023	0,395	-0,221	0,533
B1	14	0	6,631	0,353	6,365	1,322	4,600	9,310	5,713	7,387	0,933	0,676	0,122
B2	16	0	7,773	0,433	7,600	1,146	6,500	9,780	6,590	8,330	0,711	0,290	0,572
C	15	0	7,332	0,347	7,725	1,297	3,710	8,900	6,973	7,930	-1,766	4,263	0,013
FATE	13	0	7,669	0,624	7,715	2,161	4,570	11,280	5,810	9,015	0,405	-0,440	0,509
**LVFWs mm**													
Healthy	33	0	6,761	0,211	6,485	1,157	5,010	9,890	5,758	7,425	0,717	0,241	0,181
B1	14	0	8,807	0,452	8,690	1,691	5,000	11,480	7,745	10,260	-0,488	0,593	0,648
B2	16	0	8,934	0,372	8,500	0,984	8,150	10,860	8,300	9,660	1,597	1,923	0,034
C	15	0	9,196	0,332	9,260	1,241	6,900	11,470	8,460	10,055	-0,132	0,0581	0,975
FATE	13	0	9,098	0,542	9,090	1,878	6,720	13,340	7,463	10,040	0,814	1,068	0,391
**LA/Ao**													
Healthy	33	0	1,291	0,0262	1,290	0,148	1,020	1,530	1,160	1,408	0,0899	-1,015	0,210
B1	14	0	1,328	0,0325	1,325	0,122	1,130	1,500	1,215	1,435	0,0154	-1,315	0,438
B2	16	0	1,793	0,0759	1,770	0,201	1,600	2,130	1,600	1,970	0,769	-0,487	0,363
C	15	0	1,812	0,138	1,780	0,497	1,240	2,830	1,400	2,280	0,739	-0,433	0,195
FATE	13	0	1,878	0,0938	1,720	0,311	1,380	2,370	1,700	2,230	0,309	-0,873	0,323
**LAd mm**													
Healthy	33	0	5,301	1,066	1,200	6,032	0,700	15,000	1,025	12,398	0,793	-1,335	<0,001

B1	14	0	11,000	0,871	12,000	3,258	1,000	15,000	10,000	13,000	-2,382	7,373	0,001
B2	16	0	13,029	0,391	13,000	1,036	11,000	16,200	13,000	14,000	-1,224	2,597	0,064
C	15	0	15,462	0,965	14,000	3,479	12,000	24,000	13,000	17,000	1,379	1,869	0,039
FATE	13	0	17,417	0,988	18,500	3,423	12,000	24,000	14,250	19,750	0,177	-0,376	0,496
**LAV mL**													
Healthy	33	0	1,085	0,0477	1,000	0,261	0,680	2,000	1,000	1,035	1,879	4,414	<0,001
B1	14	0	1,273	0,141	1,000	0,467	1,000	2,000	1,000	2,000	1,189	-0,764	<0,001
B2	16	0	2,667	0,494	2,500	1,211	1,000	4,000	1,750	4,000	-0,0751	-1,550	0,415
C	15	0	4,154	0,715	3,000	2,577	2,000	11,000	2,500	5,500	1,802	3,413	0,004
FATE	13	0	4,111	0,716	4,000	2,147	1,000	7,000	2,500	6,500	0,206	-1,187	0,440
**FS%**													
Healthy	33	0	44,567	1,226	44,000	6,714	34,000	59,000	39,750	49,000	0,450	-0,424	0,422
B1	14	0	45,571	3,086	48,500	11,547	19,000	65,000	37,750	50,750	-0,552	1,144	0,614
B2	16	0	46,857	4,073	45,000	10,777	32,000	62,000	36,000	55,000	-0,0396	-1,170	0,817
C	15	0	45,643	3,649	46,500	13,653	26,000	74,000	36,250	50,000	0,765	0,641	0,189
FATE	13	0	38,500	3,244	39,000	11,237	18,000	55,000	30,250	49,500	-0,225	-0,557	0,885
**Mitral E/A**													
Healthy	33	4*	1,206	0,0741	1,185	0,296	0,710	1,500	1,025	1,380	0,333	-0,332	0,288
B1	14	2*	1,152	0,0995	1,100	0,223	0,900	1,500	0,980	1,350	0,958	1,575	0,699
B2	16	2*	1,230	0,0372	1,245	0,0744	1,130	1,300	1,152	1,293	-0,962	0,281	0,676
C	15	3*	1,119	0,239	1,000	0,676	0,570	2,700	0,650	1,140	2,225	5,633	0,004
FATE	13	3*	1,397	0,166	1,465	0,407	0,860	2,000	0,995	1,663	0,120	-0,288	0,826
**LV GLS% 4CH**													
Healthy	33	2**	-21,112	-0,713	-20,650	-3,637	-20,650	-15,300	-18,075	-24,275	0,313	-0,597	0,535
B1	14	1**	-16,042	-1,164	-15,500	-4,033	-15,500	-11,100	-13,625	-17,300	1,694	4,263	0,045
B2	16	0	-15,633	-2,691	-16,350	-6,592	-16,350	-7,000	-8,500	-22,275	-0,269	-1,710	0,517
C	15	0	-12,221	-0,901	-11,700	-3,372	-11,700	-6,100	-9,650	-14,175	0,445	0,633	0,917
FATE	13	1**	-12,221	-0,901	-11,700	-3,372	-11,700	-6,100	-9,650	-14,175	0,445	0,633	0,917
**LACI-ED**													
Healthy	33	0	35,288	2,431	33,300	13,750	17,000	50,000	22,250	50,000	-0,0180	-1,885	<0,001
B1	14	0	60,714	5,690	50,000	21,291	50,000	100,00	50,000	62,500	1,566	0,501	<0,001
B2	16	0	125,000	55,902	75,000	136,93	50,000	400,00	50,000	175,00	2,279	5,328	0,001
C	15	0	158,250	17,234	150,000	48,746	100,000	233,00	108,250	200,00	0,248	-1,212	0,446

FATE	13	0	133,622	32,659	80,000	97,978	40,000	300,00	50,000	225,00	0,694	-1,161	0,097
**LACI-ED**												
Healthy	33	0	35,288	2,431	33,300	13,750	17,000	50,000	22,250	50,000	-0,0180	-1,885	<0,001
Asymptomatic (stages B1+B2)	30	0	80,000	17,547	50,000	78,472	50,000	400,00	50,000	100,000	3,925	16,460	<0,001
Symptomatic (stage C)	15	0	158,250	17,234	150,000	48,746	100,000	233,00	108,250	200,000	0,248	-1,212	0,446
FATE	13	0	133,622	32,659	80,000	97,978	40,000	300,00	50,000	225,00	0,694	-1,161	0,097
**LACI-ED**												
Healthy	33	0	35,288	13,750	33,300	2,431	17,000	50,000	22,250	50,000	-0,0180	-1,885	<0,001
HCM (stage B1+B2+C)	45	0	102,357	79,027	75,000	14,935	50,000	400,00	50,000	124,750	2,319	6,645	<0,001
FATE	13	0	133,622	97,978	80,000	32,659	40,000	300,00	50,000	225,000	0,694	-1,161	0,097

*In these cases, MVE/A was not measurable owing to E–A wave fusion. **LV GLS measurements were not feasible in those cases due to suboptimal image quality.*

Supplementary Table S2. Multiple linear regression analysis of determinants of LACI-ED

Predictor	Coefficient	Std. Error	t-value	p-value	VIF
Constant	-134.699	84.636	-1.592	0.120	—
Body weight (BW)	-5.178	8.313	-0.623	0.537	1.096
Age (years)	-0.178	2.885	-0.062	0.951	1.150
Left atrial volume	11.495	8.033	1.431	0.161	1.949
Interventricular septal diameter at end-diastole	-4.911	6.583	-0.746	0.460	1.528
Left ventricular posterior wall diameter at end-diastole	9.724	6.654	1.461	0.152	1.823
Fractional shortening	0.709	0.886	0.800	0.429	1.166
Mitral E/A ratio	43.764	22.958	1.906	0.064	1.422
LV GLS (4-chamber)	3.661	2.011	1.820	0.077	1.366

In the multivariable linear regression analysis of LACI-ED, to minimize multicollinearity, only one representative parameter from each group of related echocardiographic variables was included in the model, including diastolic wall thicknesses (interventricular septum and left ventricular posterior wall), left atrial volume, fractional shortening (FS) and left ventricular global longitudinal strain (LV GLS) for systolic function, and mitral E/A ratio for diastolic function.

Supplementary Table S3. Contingency table underlying ROC-derived classification of FATE using the LACI-ED >150% threshold

LACI-ED threshold	FATE (+)	FATE (–)
>150%	6	7
≤150%	7	38
